# Use of mobile data collection systems within large-scale epidemiological field trials: findings and lessons-learned from a vector control trial in Iquitos, Peru

**DOI:** 10.1186/s12889-022-14301-7

**Published:** 2022-10-15

**Authors:** William H. Elson, Anna B. Kawiecki, Marisa A. P. Donnelly, Arnold O. Noriega, Jody K. Simpson, Din Syafruddin, Ismail Ekoprayitno Rozi, Neil F. Lobo, Christopher M. Barker, Thomas W. Scott, Nicole L. Achee, Amy C. Morrison

**Affiliations:** 1 University of California Davis, Davis, CA USA; 2 Department of Biological Sciences, Eck Institute for Global Health, Notre Dame, IND USA; 3 Eijkman Institute for Molecular Biology, Jakarta, Indonesia

**Keywords:** Mobile data collection, CommCare, Vector control, Clinical trial, Data quality, Data monitoring, *Aedes aegypti*, Dengue, Spatial repellent

## Abstract

Vector-borne diseases are among the most burdensome infectious diseases worldwide with high burden to health systems in developing regions in the tropics. For many of these diseases, vector control to reduce human biting rates or arthropod populations remains the primary strategy for prevention. New vector control interventions intended to be marketed through public health channels must be assessed by the World Health Organization for public health value using data generated from large-scale trials integrating epidemiological endpoints of human health impact. Such phase III trials typically follow large numbers of study subjects to meet necessary power requirements for detecting significant differences between treatment arms, thereby generating substantive and complex datasets. Data is often gathered directly in the field, in resource-poor settings, leading to challenges in efficient data reporting and/or quality assurance. With advancing technology, mobile data collection (MDC) systems have been implemented in many studies to overcome these challenges. Here we describe the development and implementation of a MDC system during a randomized-cluster, placebo-controlled clinical trial evaluating the protective efficacy of a spatial repellent intervention in reducing human infection with *Aedes*-borne viruses (ABV) in the urban setting of Iquitos, Peru, as well as the data management system that supported it. We discuss the benefits, remaining capacity gaps and the key lessons learned from using a MDC system in this context in detail.

**Supplementary Information:**

The online version contains supplementary material available at 10.1186/s12889-022-14301-7.

## Background

Vector borne diseases such as malaria and dengue are a major threat to global public health. They are among the most rapidly expanding infectious diseases, accounting for 17% of the human infectious disease burden, with a disproportionate burden to health systems in resource-limited low and middle income countries (LMICs) [1]. While control and prevention of vector-borne diseases will rely on integrated approaches using several strategies, such as vaccines, housing improvement and environmental management, vector control remains an underlying foundation to success. However, there is often a lack of evidence supporting the efficacy of a given vector control strategy, due to scarcity of rigorously designed large-scale epidemiological field trials [2, 3]. In its 2017 Global Vector Control Response (GVCR), the World Health Organization (WHO) set the ambitious goal of reducing the incidence of vector borne diseases by 60% in 2030 [2]. Achieving this goal will require alternative vector control tools and strategies, including the potential use of spatial repellents (SR) in public health programs. As part of its policy-making strategy, the WHO requires evidence of human-health impact for novel vector interventions from at least two clinical trials with epidemiological end-points (phase III trials) in order to assess the public health value of the intervention and determine if it should be endorsed by the WHO to be included in public health programs [2].

Optimal implementation of such large-scale, clinical trials includes rigorous monitoring of intervention coverage, study subject compliance and adverse events for accurate interpretation of efficacy, acceptability and safety of the intervention [2, 3]. Therefore, high-quality data collection remains a cornerstone of a well-conducted trial. Traditionally, data collection has relied on manual annotation on paper followed by digital data entry. This approach delays data verification and subsequently poses challenges to real-time monitoring of information and assurances of per-protocol study activity implementation.

Mobile data collection (MDC) systems, i.e., systems that use portable devices such as mobile phones or tablets for digital data collection, are used increasingly in health-related contexts and may overcome some of the challenges associated with field-based paper data collection [4–9]. In recognition of the benefits of digital health (including MDC systems) the World Health Assembly recently unanimously approved a resolution acknowledging its potential in helping meet the United Nations’ Sustainable Development Goals that specifically include vector-borne diseases [10, 11]. MDC systems have been successfully implemented in a variety of settings, demonstrating improvements in timeliness of data entry, data quality and data access [5, 6, 12–17]. Whilst their use has been described previously in clinical trials and vector control contexts [5, 18, 19], there is a dearth of information on the application and challenges in utilizing these systems in large-scale vector control field trials.

### Aims

Here we describe the development, implementation and lessons learned from the use of a MDC system in a phase III randomized-cluster, placebo-controlled clinical trial evaluating the efficacy of a SR to reduce human infection with *Aedes*-borne viruses in the urban setting of Iquitos, Peru [20]. The overarching aim is to inform health stakeholders (investigators, funders, industries, health authorities) of the challenges and advantages of implementing MDC systems in similar field trials.

## Main text

### Mobile data collection (MDC) system development

#### Motivation for MDC system and Iquitos trial context

The SR clinical trial was conducted in the city of Iquitos in the Northeastern Peruvian Amazon, which has a well-established infrastructure for studying urban *Aedes*-borne viral diseases supported by more than 2 decades of longitudinal epidemiological and entomological databases [21–24] . The city has a population of approximately 400,000 and is only accessible by boat or plane. Although internet access and cellular data coverage are available throughout Iquitos, data transfer speeds are limited, variable, and frequently interrupted. Iquitos has been the site of several vector control trials, in which paper records have been used successfully as the principal media for data collection [25–28]. During the planning phase of the SR trial and based on the previous experience of the research team, we determined that MDC could be beneficial, particularly for types of data not amenable to paper collection, due to the large scale of the study and nature of the trial endpoints. Specifically, assessment of trial endpoints required careful tracking of person-time (i.e., number of days individuals were active in the study area during the trial) and person-time covered by the intervention (i.e., number of days participants were active in the study area and had the intervention deployed in their home) by the project field staff. To calculate these metrics, it was necessary for the field research teams to be able to monitor the statuses of both the study subjects (present in the home or not) and households (intervention properly deployed or not) over time. Because study procedures related to subject follow-up and SR product replacement (that occurred every 2 weeks) were to be guided by these metrics, it was crucial that field teams had access to up-to-date information, which was unfeasible using paper-based data management methods. MDC technology provided a viable alternative to allow the field teams to monitor the daily participation status of our subjects and the proper placement of the SR product in participating households in real time. Field teams collected the data on their mobile devices directly in digital form, which were subsequently synced and displayed to project staff to inform follow-up activities. The implementation of MDC in this study required selection of a MDC platform, development, testing and piloting of MDC applications, integration of the data collected using MDC with other data sources and providing access to the collected data to all research team members who needed it. Before describing each of these steps, we provide a brief description of the Iquitos data management system and its components prior to describing the MDC components.

#### Iquitos program data management system (DMS)

Starting in 1998, our research team developed a cohort research infrastructure that allowed the linkage of human (serological, virological, clinical, and behavioural) and mosquito survey data (species-specific abundance, container habitat, and age structure) at the household level. The base component of this system was a geographic information system (GIS) developed for the city over 15 years that now contains > 70,000 of the approximately 95,000 individual lots in the city. This system was originally developed in ARC/INFO and ArcView software (ESRI, Inc., Redlands, CA), from a base map of city blocks developed from ortho-corrected 1995 aerial photographs [24], updated based on other digital maps (municipal sources) and fine-resolution satellite images. Prior to the start of the SR trial we switched from ArcView to Quantum GIS (QGIS) software [29] to manage our location data, as the location data could be more easily integrated with our PostgreSQL database through the PostGIS database extension [30]. In addition, we transitioned from geocoding the individual houses/lots as points to recording them as polygons to better track the dynamic nature of the built environment in Iquitos, where houses often split or merge to accommodate different family structures, and to allow for more precise calculations of the area of each house. Each house was assigned a location code that could be recognized by project staff in the field, which corresponded to the geolocated polygon for the house in the project database. Our project’s system of assigning location codes has been used during earlier research projects since 1998, which is why we maintained our in-house location code system based on polygons rather than adopting grid-based address systems that were made available in more recent years such as Google’s Plus Codes [31] or What3words [32]. Continued improvements in technology and the availability of open-source platforms make development of a GIS for any project more practical and feasible.

Location data was managed together with data from other sources (such as study participant data and entomological data) in an integrated data management system (DMS) that was upgraded using Django [33], a Python web-framework that followed a model-view-template architecture. The DMS included a secure PostgreSQL database linked to our Django web interface that we developed and built with open-source software and three 64-bit database servers (1 TB storage, 8 GB RAM). Secure servers were housed in our two laboratory facilities in Iquitos and in a secure server facility on the University of California (UC) Davis campus. High-speed communication between the servers allowed for a constant data flow, maintaining the same data on all three servers. This allowed for high-speed access to data at UC Davis for team members based in the U.S. and significantly increased security due to the redundancy of the offsite data backup. Data access and sharing were mediated through our secure website and limited to authorized users.

Lots in the SR study area were predominantly areas associated with individual houses, but some included churches, small businesses (carpentry, vehicle repair, sewing), restaurants, offices, and vacant lots. Apart from schools, hospitals, and some offices, most lots contained a single structure, sometimes with a separate bathroom or storeroom. Housing was very dense, so most lots shared walls and had backyards separated by brick or cement walls. Many houses in Iquitos had multiple families sharing homes, sometimes with delineated living spaces, but more often with shared spaces. These family and housing structures in Iquitos changed frequently over time and required a flexible system to keep track of both the changes in the built environment and in the location of residence of each participant. For the SR trial, lots were defined by the presence of a front door, clear side and back wall. The DMS facilitated the addition of new locations, either because they were newly registered locations, or represented houses that divided into two residences, or multiple houses that merged into one. Each new house/lot was assigned a “location code” and an “active date” to record changes in housing structure throughout the study. Lots could be updated by (1) assigning an “inactive date” to an existing house and (2) redrawing new polygons for houses that were changed, assigning a new activation date and location code for each (often adding/removing alphanumeric suffixes). Active and inactive dates defined the beginning and ending dates for each house in the study, and at any time during the study, houses that were active could be identified easily as those lacking inactive dates (i.e., houses with null values for inactive dates).

Each individual participant was registered in the DMS through a “census” form for an individual house/lot, and all individual data were geocoded to the house level in the GIS database. Individuals in a house (that had to exist in the GIS) were assigned a “participant_code” that included the “location_code” and a suffix representing the individual. For example, five people enrolled at the location_code MYC200 would have been assigned the following participant_codes: MYC200P01, …, MYC200P05. If people moved or changed houses this information was managed in the “participant_status” table. Changes to participant’s statuses were tracked over time by including a start and stop date corresponding to each participant code. Active codes were identified as those without a stop date at any given time. This information was used to calculate the person-time each individual was under surveillance during any time interval specified. Updating of this status information was done using the MDC (described below) facilitating updates of status data in real time. The most innovative aspect of this data structure was the ability to follow individuals who moved between houses, spent time in multiple houses simultaneously, or were lost to follow-up during the study.

All information collected for a human participant was grouped through a “consent” table linking individuals to different components or levels of participation in the study. Examples from the SR trial include routine febrile surveillance (regular visits from study staff 3 times per week to inquire if anyone in each household is ill), acute febrile illness (paired acute and convalescent blood samples following clinical illness), and enrollment in the longitudinal cohort (annual blood samples for serological testing to identify individuals who were infected during the preceding interval). The consent ID (identifier) then linked to samples and their laboratory results and clinical data. All entomological surveys were linked through the location code.

The data management strategy and GIS described above, received Institutional Review Board (IRB) and Regional Health Authority (DIRESA) approval for seven large cohort studies carried out between 1999 and 2019 in addition to the SR study. All procedures comply with US Federal and Peruvian regulations governing the protections of human subjects. Our studies have monitored as many as 20,000 human participants at the same time and required that field staff could identify individual participants and households over time with no errors or confusion. Our DMS, which included personally identifiable information (PII), was critical for proper management of the study, and the system was available only to authorized study staff with appropriate human-subject training.

#### MDC platform selection

A number of different platforms exist on which MDC systems can be developed [6, 34]. For our trials, we used CommCare (Dimagi Inc., Cambridge, Massachusetts, USA) because of features that were well-suited for our project, including case management, ability to develop custom surveys without the prerequisite of coding skills and drop-down response options to enable built-in constraints for data quality control, among others. Perhaps most relevant to our Iquitos trial, the ‘case management’ feature enabled tracking units of interest (cases), such as people or houses, over time, which is invaluable for longitudinal studies as subject and house status may change frequently during the follow up period. Once a case is registered, all questionnaires (forms) associated with that case are linked by a unique ID ensuring all changes to cases can be monitored. Key information associated with a specific case can be viewed by field staff on mobile devices at the time of follow-up [35, 36].

CommCare allows edits to the data collection structure (modifications to the survey forms) as well as the collected data (modification of values entered for a given form) using the web-based application. Crucially, CommCare logs these changes such that they can be tracked, maintaining an audit trail of modifications for assurances of good clinical practices. However, the error editing mechanism on the web-based platform is not designed for bulk edits, making these burdensome.

In addition, CommCare servers on which data are stored are secure and transmitted data are encrypted. Data access requires authentication and, if desired, two-step authentication can be used to further enhance data security. Data can be accessed directly by downloading comma-separated-value (CSV) files from the web application, or by extraction through CommCare’s advanced application programming interface (API).

Lastly, important to large-scale trial management, CommCare provides an automated reporting system, where data summaries such as individual field staff activity (e.g., number of data forms completed) can be forwarded to project managers periodically by email to facilitate oversight.

One of the primary limitations of using a cloud-based mobile data collection platform is that data must be synchronized regularly from mobile devices to centralized, cloud-based servers if the data entered by one user is to be available to all other users of the application. When multiple individuals are working in the same team this is particularly important. This would be an insurmountable obstacle in trial settings where regular extended internet outages cannot be avoided.

#### Application development

We developed two applications for our MDC system using the CommCare platform: 1) an intervention management application (IM-app) to monitor SR intervention initial deployment, replacement, and removal and 2) a subject management application (SM-app) to monitor house febrile illness surveillance visits, census updates and adverse events (Supplementary Table 1). The principal objectives of these applications were to empower field teams to carry out their work more effectively and efficiently by providing them with near-real-time data summaries of their assigned houses or subjects (e.g. whether a particular house was due to have a febrile surveillance visit or the intervention replaced) and to allow the accurate measurement of person-time at risk from census updates. Combined, the two applications facilitated rigorous calculation of person-time under protection to better interpret our trial outcome of protective efficacy.

**Fig. 1:**
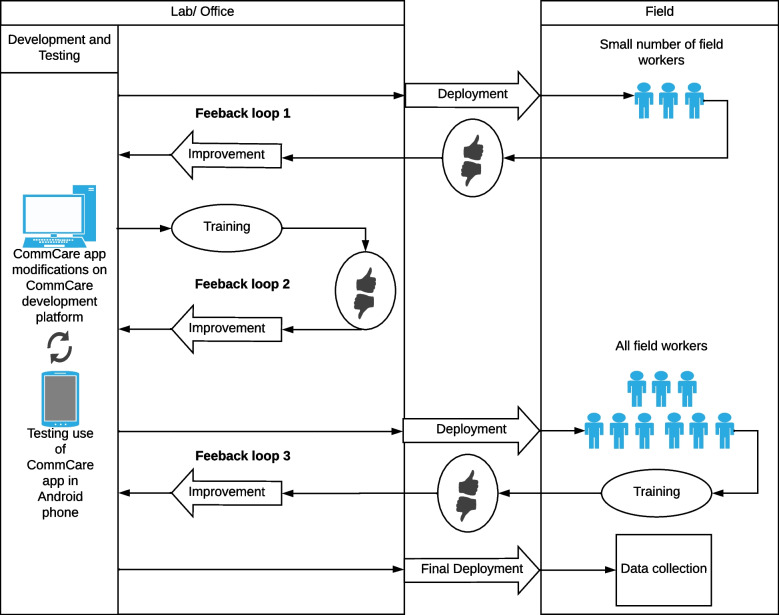
MDC application development and optimization: 1) Initial development; 2) Pilot test in the trial environment by a reduced number of field workers (first feedback loop); 3) Hands-on demonstration and training in the lab/office (second feedback loop); 4) Training and beta-testing in trial environment (third feedback loop); 5) Final deployment

The framework for application development and improvement adopted in the Iquitos trial is outlined in Fig. 1. Initial development and testing of the MDS occurred at our field laboratory in Iquitos. Development of the application was approached through an iterative process of application testing followed by improvements based on user feedback, that can be described through three types of feedback loops between field teams and application developers: Loop 1) a pilot version tested in the field by a small number of senior field staff; Loop 2) all field staff participating in hands-on application training; and Loop 3) beta-testing by all field staff for final optimization. A MDC system ‘clinic’ was hosted each day for field workers to troubleshoot problems with application developers. This service was available during and after the application development, although this became more informal as users became familiar with the applications. Each app was developed separately and was used by a different field team, therefore the testing and training of the field staff was independent for each of the apps. However, both apps followed the same general framework for development and improvement.

#### Data integration, validation and access

**Fig. 2:**
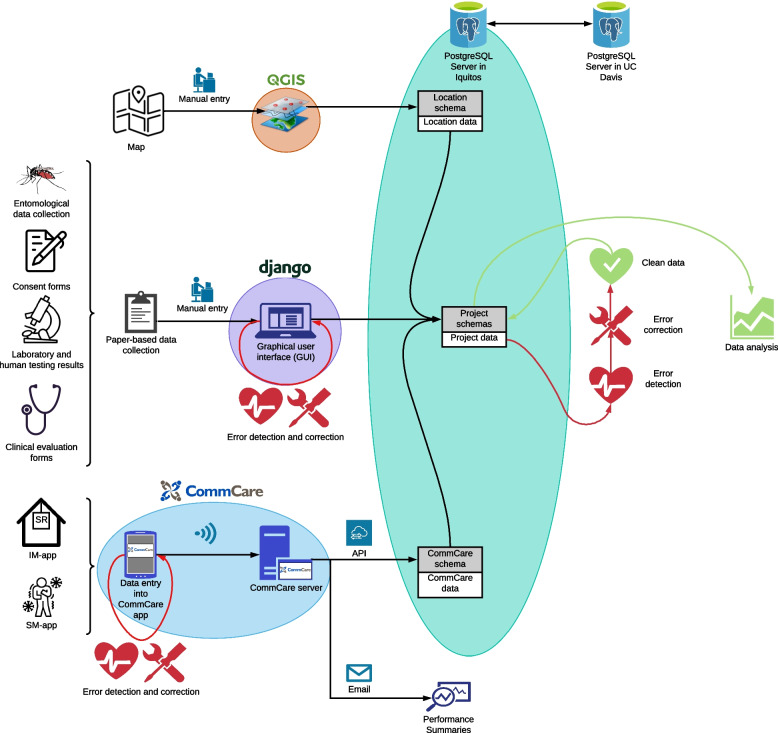
Overview of data flow, validation, and integration framework

The overarching framework for data flow is summarized in Fig. 2. The body of Iquitos data encompassed different sources, forms of data collection, and formats, making integration a challenge. Because each house in Iquitos was encoded in a GIS with spatial coordinates and an alphanumeric code [24], field teams were able to record them easily from provided maps as well as those codes painted on the front of each house, and QR tags that were placed on the back of each door such that the code was visible and easily scanned by our mobile devices. Similarly, study participants were identified based on the alphanumeric code of their main residence. Critical to managing the SR trial was having a flexible system that could track changes in houses and the location of human participants.

The spatial database and other project data were housed in a PostgreSQL server with the PostGIS extension that allowed the storage and integration of spatial and non-spatial data types. Project data stored in the PostgreSQL server included historical data and data collected using paper forms (such as entomological data, laboratory results, participation consents) that were input into the database using a web-based data entry graphical user interface (GUI), developed in Django [33]. Manual input of paper forms remained necessary for certain aspects of the project that required a physical format, such as a signature from a study participant on a consent form, a biological sample, or entomological survey analysis (determination of the species, sex, and number of mosquitoes) in the lab. While some of these data sources could also potentially be digitized, that was not prioritized for this project. Instead, a system of barcode stickers associated physical study components with participant or location data. Results from laboratory testing were often produced directly by laboratory instruments, requiring technical expertise to be imported and reformatted into a usable structure in the database. Integration of the CommCare data with the PostgreSQL server occurred through the CommCare application programming interface (API), a process that required programming expertise (Fig. 2).

Data integrity checks occurred at multiple points along the data pathway (Fig. 2). Within the MDC system, skip logic (i.e., skipping of questions as applicable based on form responses) and CommCare case management functionality was embedded during development to constrain data entry options and thereby incidental errors. Data variable thresholds and rules were also applied for added quality control, preventing nonsensical values from being entered (e.g., birthdates in the future). These integrity checks were only possible thanks to the digital nature of data collection using the mobile devices, and greatly reduced errors associated with free-hand entry of values and with manual data entry into the database, as well as reducing data loss associated with physical data collection, such as misplacement of forms or illegible writing. In addition, weekly data summaries of blinded data were assessed by data management staff for near-real-time error resolutions and cleaning. These data summaries were easily produced due to the immediate availability of data collected in digital form, allowing for the timely integration with the remaining project data not collected using MDC. Data integrity checks were also performed during non-MDC project data entry to ensure accurate relations among data for unique identifiers (e.g., location code matched to entomology and/or blood results).

Utilizing the PostgreSQL server as a single access point for all project data greatly facilitated data validation. A GIS system that allowed synchronisation between our spatial and relational databases was also crucial. Code was written in SQL and R languages to query and correct data inconsistencies, most commonly consisting of errors in the house location codes. This approach facilitated updating and correcting the CommCare applications based on changes in the project data (for example, updating the location code for a certain location to reflect changes in house structure). For errors that were not corrected programmatically, data management staff communicated with field teams for re-collection in the field. This was only possible because integrity checks were performed at regular intervals during the lifetime of the project. Having timely access to data collected in the field in digital form through the MDC platform allowed close-to-real-time data validation and error correction.

### MDC system in practice

#### Staff activities: monitoring and trial implementation

Field workers were assigned to one of two primary activities, intervention management (IM-team) or subject management (SM-team). The IM-team consisted of 20 entomological field staff divided into two groups of 10 people, with each group responsible for managing 13 project clusters with a median of 156 houses present in each cluster (Interquartile range [IQR] 142–168). One individual in each group was dedicated to mobile data entry using an Android mobile device (either a tablet or cell phone). This individual used the IM-app to determine and record the number of intervention units applied inside each house at initial deployment [based on manufacturer specifications of target density (one unit per 9 m^2^)] and record the timing of intervention deployment, replacement, or removal (Table 1). The SM-team included 35 nurse-technicians, each with a mobile device, who used the SM-app to record census updates from 4039 houses and outcomes from routine subject febrile illness surveillance to identify potential dengue or Zika virus infection [37]. Both the IM and SM teams were responsible for reporting indicators of potential adverse events in subjects and family members of enrolled houses.

**Table 1:** Total number of uploaded forms per month during the Iquitos, Peru trial and median time to completion for data entry using each form

App	Form	Total No. forms	Median forms/month (IQR)	Median form completion time in seconds (IQR)
SM	Surveillance visit	297,983	14,562 (11,189-19,384)	7 (6–12)
IM	Change	105,493	3710 (2983-4160)	8 (6–11)
SM	Census	9267	422 (81–524)	97 (71–144)
IM	Calculator	3037	28 (17–56)	32 (10–91)
IM	Removal	2804	26 (20–52)	6 (4–16)
IM	Deployment	2432	36 (20–58)	96 (59–156)
SM	Adverse event	129	3 (2–23)	214 (130–321)

Forms completed each day were synchronized and data uploaded to the CommCare server at the project headquarters in Iquitos. This allowed up-to-date information to be available to both project managers and field teams, allowing them to monitor progress of field operations and maintain data quality control. Project managers received regular reports regarding field activities through CommCare’s automated report system or through reports compiled by data support workers using CommCare’s basic data access tools. For example, for the IM-team we created a twice-monthly report of the percentage of houses that had successful intervention replacement that met required product coverage. This enabled activity management for field teams and data-driven responses to improve intervention coverage. Similarly, CommCare’s reporting system was utilised to inform project supervisors of field-worker activity to ensure subject surveillance efforts were followed up and carried out in households where unsuccessful visits were reported. Ultimately, we were able to document appropriate SR product coverage in 81% of registered houses throughout the trial, which required product replacement every 2 weeks. Our SM team averaged 2.5 visits per house per week for the duration of the study allowing documentation of daily subject participation status. Field workers were able to view updated information displayed on the screen in their data collection application, such as the last time the intervention was deployed in that household, or the study participation or verification of household census, allowing them to make on-site decisions.

#### Collection of geolocation data

As described above, houses in the study area were encoded as polygons in a GIS system with coordinates and an alphanumeric location code that also related to the participant code for the study subjects residing in each location. Field teams recorded the location code associated with the IM or SM data using printed maps, QR codes on the house door that provided the location code, or location codes painted on the front of the house. These methods for recording geolocation data were used instead of the in-app CommCare GPS capabilities, given that GPS data often lack the accuracy and precision needed to distinguish between houses in densely populated urban settings [38, 39].

In Iquitos, we described the dynamic nature of housing, and tracking these changes was always a major challenge and source of data incongruencies in the study. Changes in location codes needed to be updated in the field (repainted, QR tags replaced), as well as in the GIS database, where polygons had to be re-drawn and assigned updated codes. These activities required considerable staff effort and communication between the field teams and data managers. A crucial component of the MDC was the census update survey in the SM-app, which provided a mechanism to update house and participant status information in near real time, flagging when changes to house codes or participant residences needed to be made. When changes were not updated in a timely manner, the different teams assigned data to outdated or diverging house-codes. Many errors also resulted from the initial decision to develop two separate apps (the IM-app and the SM-app) to collect data in the same houses, as often the house location codes were different for the same house in the two different apps. The method of recording house location code by the field teams also affected the number of errors: using the CommCare case management tool to record the house location code by providing a dropdown list of “active” locations instead of inputting the code manually in a free-text field reduced house code errors in the SM-app by more than half, from an average of 175 to 77 errors per month. However, by using this functionality, when field workers encountered houses with codes that did not appear in their list, they were unable to collect the data, making timely updates of changes to the location codes in the case list, as well as the GIS database, crucial for data quality. In our opinion, tracking of the information collected in the census update form in the MDC, combined with rapid communication with the GIS team, provided a mechanism to make those updates that would not have been possible without the mobile application.

#### Time burden for data entry and synchronisation

Data collected by field teams were input directly into mobile devices in the field and later uploaded into the cloud using the broadband internet connectivity at project headquarters. Because of the high volume of data required for capture by field teams, MDC forms were designed to be completed quickly and efficiently. Table 1 outlines the form volume during the trial and the median time for form completion. The most frequently completed forms were the illness surveillance and the intervention change forms. It is notable that these forms had a median completion time of less than 10 seconds.

The high volume of data collected also affected the timeliness of data synchronization with the cloud, which in turn affected information being available in real-time to adjust project activities. The timeliness and reliability of synchronization was improved by reducing the number of cases, switching the case follow-up unit from individual SR intervention products (of which there were approximately 24,000 at any one time) to individual houses (of which there were approximately 4000 at any one time) where the products were placed.

## Conclusion

Considerable resources are required to design, implement and manage high-quality data collection systems that capture epidemiological outcomes from vector control trials. Mobile applications for digital data collection of health research data have gained substantial attention over the past decade as a tool to increase data collection efficiency [4–9]. These systems tend to be relatively simple to develop and maintain, and they minimize the time between initial data collection and reporting of data to researchers and stakeholders. The use of a CommCare MDC platform described here afforded study teams the ability to integrate and manage large, complex datasets through the support of computer coding-competent researchers rather than dedicated software developers.

While MDC can be extremely useful in some settings, it is important to note that not all studies will benefit to the same extent from using an MDC system. For example, a clinical trial conducted in Sumba Island, Indonesia to evaluate the same SR product against malaria [40], with similar needs of tracking product replacement and epidemiological and entomological follow-up data, consisted of study clusters located within remote areas without internet connectivity. Due to the lack of connectivity infrastructure, data were collected on paper and housed in a different location from where most of the data managers and programmers were based. This approach required an extra data entry step to input data into the MDC platform where it was accessible to programmers for data quality checks. This delayed the data validation process such that it did not take place until late in the study, making it difficult to correct many of the identified errors on-site. By that time, the number of errors was so large that manual correction was impractical and instead data validation became a technical, labor-intensive process that required help from programmers. This process also complicated data access for project analysts and statisticians. In addition, by not making it an immediate process, it was not as useful for monitoring project activity performance. In this context of lack of connectivity and physical separation between the field teams, the developers and the project leads, MDC systems offer few advantages over paper collection.

Below we provide key recommendations and lessons learned about MDC systems specifically from our experiences in Iquitos.

**Table:** 

**Summary Box 1.** Key lessons learned using a MDC system in vector control clinical trials. ***Lesson 1: Mobile data collection should not stand alone*** ***Lesson 2: Mobile data collection is best suited for relatively quick field-based tasks that are repeated frequently and need to be available in close to real-time*** ***Lesson 3: Simple mobile data collection applications can be an advantage in resource-poor settings*** ***Lesson 4: Mobile data collection systems, as well as the data management systems that support them, require the presence of coding-competent personnel based in the field site. This allows effective piloting of applications, training of the field teams collecting the data, and maintenance and support of the application use.*** ***Lesson 5: Blend strategies to optimise the balance between data integrity and user experience***	

### Lesson 1: Mobile data collection should not stand alone

MDC is an important tool for execution of vector control or other types of clinical trials. However, trials in urban settings and with epidemiological and/or entomological endpoints will require MDC to be a component of a larger functional data management system (DMS) capable of integrating data from MDC and non-MDC sources. Recording location data in dense urban settings will require a previously developed GIS system that can manage geo-referenced locations, given the inaccuracy of GPS location data in these settings [38, 39]. Entomological and epidemiological study endpoints will often include results from laboratory testing, and studies with human participants will also need to associate consent forms and other paper-based forms with other participant information, laboratory results, and locations. Integration of data collected from different sources (MDC, paper-based forms, laboratory testing, GIS, etc.) is best done using a relational database. Developing these data management systems and integrating MDC-collected data with them represent a considerable investment and require technical expertise but should be a requirement for these types of trials.

Thus, for the SR trial, there were two prerequisite DMS components that were essential. First, a pre-existing set of geographic coordinates for our study clusters that were part of a flexible GIS. In addition to allowing for accurate collection of geolocation data, a key advantage of our GIS system was the flexibility afforded by using of polygons with active and inactive dates, allowing us to record changes in the built environment over time. Furthermore, a system for house code identification with redundancies, in our case painted codes and QR tags, is required. We strongly recommend that any large-scale house-based vector control trials, especially in urban environments, have a functioning GIS in place prior to trial initiation. Fortunately, since the development of our GIS system in 1998, there are many new tools are currently available to facilitate GIS development, including access to satellite maps and open-source coding systems (Google’s Plus Codes, What3Words, etc.). Second, in our study we were able to consolidate data collected using the MDC applications with all other project data in a single PostgresSQL database that allowed the integration of spatial and non-spatial data. In our experience, it is unlikely that all project data will be collected or managed solely using mobile applications, thus, we strongly recommend integrating data collected through mobile applications into a relational database.

Developing a framework with a single point of access to project data simplified data validation, management, and access. Integration of different project data sources and enabling secure access to data was a constant challenge and required a significant amount of technical expertise. This proved to be a worthwhile effort because it enabled project analysts and statisticians direct access to all updated project data using most commonly used statistical software packages, and allowed better monitoring of access from a data security perspective. Data access should be flexible but restricted to minimise data governance and security risks.

### Lesson 2: Mobile data collection is best suited for relatively quick field-based tasks that are repeated frequently and need to be available in close to real-time

Data that can be collected or entered in a static office or lab setting, that requires lengthy qualitative input, or that does not require mobility, may be less suited for collection using mobile devices. However, the SR trial had multiple data streams that were ideally suited for MDC and for which paper collection had been impractical in the past. For example, registering individual illness surveillance for each household, only required 1) an accurate drop-down list of “active” houses, 2) a few additional clicks to register contact with a resident or 3) register a reason the visit was not successful from a predetermined list. This process was short, carried out 3 times per week, and repeated hundreds to thousands of times per month. Using the MDC system, forms tended to be completed in seconds or minutes and were quickly available for routine monitoring of staff performance by the project managers and to identify houses lost to follow up. Similarly, SR product replacement only required registration and a few additional pieces of information carried out twice per month. Finally, the census update application was only used to report changes in the existing census, thus like the previous examples a few clicks to register no change, but then the ability to add changes in participant status which flagged the need to update data in the DMS. This was crucial for quality control since any changes in the built environment required visits to the structures, validation of the changes, and subsequent updates to the GIS and to the location code recognition systems in the physical location (QR codes, painted codes on front of the house). Similarly, changes to a participant’s residence also had to be recorded in the database. Access to this table in real time greatly facilitated GIS and person-time maintenance and ensured that appropriate house codes were available in dropdown lists.

### Lesson 3: Simple mobile data collection applications can be an advantage in resource-poor settings

As MDC systems often leverage the power of cloud data storage, they may be heavily dependent on local network infrastructure and/or data access and protection policies. Iquitos is probably representative of many low- or middle-income countries internet coverage; it exists but is slow and unstable, resulting in unreliable data synchronisation. Certain functionalities can put an extra burden on the network, such as the use of CommCare’s case management feature where the case data is both uploaded and downloaded from mobile devices to the server. The CommCare documentation recommends limiting a mobile device’s case load to 1000 cases in a given application [41], highlighting the importance of choosing an appropriate case follow-up unit. For both the SR product replacement and illness surveillance applications, we originally wanted the cases to defined as individual SR products or individual participants, respectively. This made synchronisation of data unmanageable because there were approximately 24,000 products deployed to the field at any one moment and we had > 20,000 residents censused. We were subsequently forced to change the case follow-up unit to houses, of which there were approximately 4000 at any one time, improving synchronization reliability. Researchers need to remember they cannot have it all and recognize the limitations of MDC. In the case of our sister trial in Sumba Island, Indonesia, implementation of a fully functional MDC system was impossible due to unreliable network connectivity [40]. Adequate personnel and expertise tasked with maintenance of the MDC system and downstream workload associated with data cleaning and preparation for analyses, as well as infrastructure needs, should be considered during study planning and resource allocation. Clearly setting out the objectives and requirements of the MDC system at the start of a project will allow a more accurate estimation of the costs, personnel and infrastructure needed to effectively implement the system.

### Lesson 4: Mobile data collection systems, as well as the data management systems that support them, require the presence of coding-competent researchers based in the field site. This allows effective piloting of applications, training of the field teams collecting the data, and maintenance and support of the application use

Development of MDC applications is not a linear process and should be carried out in partnership with the developer, the users and project managers. In Iquitos, the presence of on-site application developers allowed them to seek frequent feedback from team members and allowed training opportunities with the field workers at various stages of the application development, in addition to providing a troubleshooting resource for field staff for the duration of MDC application usage. Also critical was the ability of developers to accompany field team members during the normal activities in the field to provide real time resolutions to questions or problems. This constant feedback loop gave local field workers many opportunities to drive MDC application development directly, thus improving data quality and simplicity of use, and meeting worker’s needs. In addition, building local capacity was regarded as a priority and this feedback loop provided an excellent opportunity to do so. Bringing members of the application development team together with end users early in the project requires a considerable investment of time and resources. Piloting of the application should include all team members to verify the application behaves as expected for everyone. In addition, analysts should check early on that the data generated by the application meets their needs.

### Lesson 5: Blend strategies to optimise the balance between data integrity and user experience

While maintaining the accuracy of the data can be a constant challenge, using strategies such as a unified data collection and storage system, using restricted value entry fields in the data collection forms and performing routine checks on the data integrity throughout the data collection activities can minimize errors. In implementing these strategies, it is important to balance flexibility for users with quality control measures to reduce errors. In our trial, we achieved a significant error reduction by re-designing the SM-app from a form-based system where the alphanumeric house location code of the survey location was recorded using a free text field to a system that used CommCare’s case management feature. The case management feature allowed field workers to choose from a list of possible pre-registered codes to select the house where the survey took place, and all survey forms were associated with that case. We strongly recommend that house-based trials provide and restrict data entry to “active” locations within the application even at the expense of delaying data collection when location data requires regular updating. Taking this measure reduced the time needed for cleaning of data and enabled us to accurately monitor and report febrile surveillance events in near-real-time. The simple step of using a dropdown list of “active” house codes that was used by both the IM-app and the SM-app was critical but debated, as data could not be collected if the house code did not appear in the list, and establishing a new code required involvement of a supervisor. However, the increase in data integrity far outweighed this inconvenience.

## Summary

The use of a MDC system in the Iquitos, Peru trial had many advantages. We found that despite the significant challenges, MDC systems provide an opportunity to enhance the integrity of the data collected during a large-scale trial in an urban resource-poor setting by reducing data entry errors, allowing timely data collection, and facilitating real-time data access. While development of MDC systems without access to advanced technical expertise has become increasingly possible, not all data collection in a vector control trial can realistically be carried out in this way. A candid evaluation of the needs that can be met using a MDC system given the resources, infrastructure and technical expertise available should be performed during the initial planning phase before embarking on such a project. Access to real-time data in large scale trials allows project members to streamline logistics during project implementation and make data-driven decisions regarding outcome interpretation through assurances of data quality. The use of MDC system technology is anticipated to facilitate assurances of trial rigor required by WHO for assessing public health impact of a candidate vector control intervention and to guide policy development. It would be remiss to not highlight that MDC systems are tools that can facilitate efficient project implementation, but not in the absence of the other fundamental components needed for successful research, such as optimal study design, a skilled research team and good leadership.

## Supplementary Information

**Additional file 1: Supplementary Table 1.** Iquitos, Peru SR Trial CommCare spatial repellent intervention (SR) and study subject surveillance (PS) application structures with data form type, function and key measures/variables.

## Data Availability

Deidentified data sets are available upon request to the corresponding author according to the program funder data access and sharing policy.

## Abbreviations

LMICs: Low and middle income countries
GVCR: Global Vector Control Response
WHO: World Health Organization
SR: Spatial repellent
MDC: Mobile data collection
DMS: Data Management System
GIS: Geographic information system
UC: University of California
ID: Identifier
IRB: Institutional Review Board
PII: Personally identifiable information
CSV: Comma-separated-value
API: Application programming interface
IM: Intervention management
SM: Subject management
IQR: Interquartile range
GUI: Graphical user interface
PE: Protective efficacy
SQL: Structured query language
