# Supplementary material for: Use of mobile data collection systems within large-scale epidemiological field trials: findings and lessons-learned from a vector control trial in Iquitos, Peru
Source: BMC Public Health. 2022 Oct 15;22:1924. doi: 10.1186/s12889-022-14301-7 (PMC9571464; doi:10.1186/s12889-022-14301-7)
Supplement: Supplementary file 1 — Additional file 1: Supplementary Table 1. Iquitos, Peru SR Trial CommCare spatial repellent intervention (SR) and study subject surveillance (PS) application structures with data form type, function and key measures/variables. [file 12889_2022_14301_MOESM1_ESM.docx]

**Supplementary Table 1**: Iquitos, Peru SR Trial CommCare intervention management application n (IM-app) and subject management application (SM-app) structures with data form type, function and key measures/variables.

| **Intervention Management (IM-app)** | | |
| --- | --- | --- |
| **App purpose:** Tracking of intervention deployment, replacement, changes and removal. Provides key logistical planning data to SR-team and enables estimation of household and cluster intervention coverage as well as time under protection. | | |
| **FORM** | **FUNCTION** | **KEY MEASURES/VARIABLES** |
| Calculator | To determine the number SR-interventions required per house | - Total area - Number of interventions needed |
| Deployment | To record deployment of interventions in houses | - Date of deployment - Number interventions deployed - Room in which deployed |
| Change | Records replacement of interventions every 2 weeks | - Date of replacement - Number of interventions replaced - If not successful why not |
| Removal | Registers removal of shield interventions | - Date removed - Reason for removal |
| **Subject Management (SM-app)** | | |
| **App purpose: 1)** Tracking of household membership to facilitate accurate calculation of person time at risk of disease. 2) Recording of house to house illness surveillance visits to enable capture of disease cases. | | |
| **FORM** | **FUNCTION** | **KEY MEASURES/VARIABLES** |
| Census | Recording household membership | - Date household update - Update of existing study subjects’ / addition of new study subjects’ time-in-house status |
| Illness_surveillance | Recording febrile surveillance visits | - Date of visit - Result of visit - Field-worker |
| Adverse event | Recording of adverse events | - Date event - Nature of event - Follow plan |
